# RNF8 is responsible for ATRA resistance in variant acute promyelocytic leukemia with GTF2I/RARA fusion, and inhibition of the ubiquitin–proteasome pathway contributes to the reversion of ATRA resistance

**DOI:** 10.1186/s12935-019-0803-4

**Published:** 2019-04-04

**Authors:** Wenzhe Yan, Ji Li, Yang Zhang, Yafei Yin, Zhao Cheng, Jiayi Wang, Guoyu Hu, Sufang Liu, Yewei Wang, Yunxiao Xu, Hongling Peng, Guangsen Zhang

**Affiliations:** 1 Department of Hematology, The Secong Xiangya Hospital, Central South University, Changsha, 410011 Hunan China; 2 Department of Oncology, The Secong Xiangya Hospital, Central South University, Changsha, 410011 Hunan China; 3 Department of Hematology, Xiangtan Central Hospital, Changsha, 410011 Hunan China; 4 Department of Nephrology, The Secong Xiangya Hospital, Central South University, Changsha, 410011 Hunan China; 5 Department of Hematology, Zhuzhou No.1 Hospital, Zhuzhou, 410011 Hunan China

**Keywords:** APL, GTF2I-RARA, RNF8, RARA, Proteasome inhibitor

## Abstract

**Background:**

*GTF2I*-*RARA* is a newly identified *RARA* fusion gene in variant acute promyelocytic leukemia (APL) patients with t(7;17)(q11;q21). Clinical manifestation in the patient showed that it is a sort of ATRA-insensitive oncogene and is different from the classic *PML*-*RARA* in terms of therapeutic reaction.

**Methods:**

To reveal the functional characteristics and regulating mechanism of the *GTF2I*-*RARA* fusion gene, we established a *GTF2I*-*RARA*-transfected HL60 cell model and examined its sensitivity to ATRA by western blot, MTT assay, flow cytometry, and Wright-Giemsa staining. Coimmunoprecipitation and confocal microscopy were used to examine the binding of GTF2I-RARA and transcriptional corepressors. We also performed ChIP-seq to search for potential target genes. Immunoprecipitation, ubiquitination assay, western blot, luciferase assay, and real-time PCR were used to analyze the effects of RNF8 on RARA. Flow cytometry and Wright-Giemsa staining were used to study the effect of MG132 and ATRA on the *GTF2I*-*RARA*-transfected HL60 cell model.

**Result:**

We confirmed resistance of GTF2I-RARA to ATRA. Compared with *PML*-*RARA, GTF2I*-*RARA* has a higher affinity to HDAC3 under ATRA treatment. Using the ChIP-sequencing approach, we identified 221 *GTF2I*-*RARA* binding sites in model cells and found that the RING finger protein 8 (RNF8) is a target gene of *GTF2I*-*RARA*. RNF8 participates in disease progression and therapy resistance in APL with the *GTF2I*-*RARA* transcript. Elevated RNF8 expression promotes the interaction between RARA and RNF8 and induces *RARA* Lys-48 linkage ubiquitylation and degradation, resulting in attenuated transcriptional activation of *RARA*.

**Conclusion:**

Our results suggest that RNF8 is a key *GTF2I*-*RARA* downstream event. Using the combination of MG132 and ATRA to treat *GTF2I*-*RARA*-HL60 cells, a synergistic effect leading to *GTF2I*-*RARA*-HL60 cell differentiation was confirmed. Taken together, the targeting of *RNF8* may be an alternative choice for treatment in variant APL with *GTF2I*-*RARA* fusion.

**Electronic supplementary material:**

The online version of this article (10.1186/s12935-019-0803-4) contains supplementary material, which is available to authorized users.

## Background

Acute promyelocytic leukemia (APL) is a distinct subtype of acute myeloid leukemia that is mostly driven by the chimeric oncoprotein *PML*-*RARA* [1]. Accruing evidence indicates that *PML*-*RARA*-driven APL is sensitive to both *all*-*trans* retinoic acid (ATRA) and arsenic trioxide (ATO) therapy [2, 3]. The combination of ATRA and chemotherapy or ATO dramatically improves the prognosis of APL. Key elements of *PML*-*RARA*-induced leukemogenesis have been elucidated. Generally, *PML*-*RARA* exhibits a high affinity for the corepressor proteins N-CoR and SMRT, and only the introduction of pharmacological doses of ATRA (1–2 μM) induces corepressor release and coactivator recruitment, as well as the degradation of *PML*-*RARA* [4, 5]. *PML*-*RARA* also acts as a transcriptional repressor of *RARA*- and non-*RARA*-target genes, which are involved in processes such as differentiation, apoptosis, blocking promyelocyte differentiation, and contributing to the proliferation of leukemic blasts [6, 7]. However, the extent to which the *RARA* fusion protein acquires altered DNA-binding capacities that may result in the aberrant expression of genes normally regulated by wild-type *RARA* is still not well understood. Hoemme et al. [8] identified a total of 372 target genes of *PML*-*RARA* using chromatin immunoprecipitation (ChIP)-on-chip. Subsequent genome-wide studies conducted by Martens et al. and Wang et al. identified nearly 3000 binding sites of *PML*-*RARA,* suggesting that *PML*-*RARA*-associated epigenetic alterations and regulatory mechanisms are very sophisticated [9, 10].

There is a subset of patients with variant APL in whom t(15;17) cannot be detected, either at a cytogenetic or molecular level. To date, 13 variant fusion genes in APL have been identified and reported [11–13]. Clinically, patients with variant APL manifest different clinical or laboratory phenotypes and treatment outcomes [14]. All variant APL cases show the same breakpoint within the *RARA* gene, whereas their partner genes are variable. Therefore, the nature of the *RARA* partner has a decisive impact on the disease phenotypes and therapeutic response to ATRA and ATO.

We have previously identified and reported a novel fusion gene *GTF2I*-*RARA* (GenBank no. KP100665.1) in a variant APL patient with cryptic t(7;17)(q11;q21) [15]. Like other *RARA* fusion genes, *GTF2I*-*RARA* shares a common *RARA* portion and acts as a dominant-negative regulator in *RARA/RXR* pathways. Our case manifested a high leukocyte count and was resistant to retinoic acid differentiation induction and chemotherapy attempts [15]. In this study, we show that a cell line harboring the *GTF2I*-*RARA* transcript is resistant to ATRA. Using ChIP-sequencing (ChIP-seq) technology, we screened and identified 221 binding sites of *GTF2I*-*RARA* and focused specifically on the RING finger protein 8 (RNF8) gene. We found that RNF8 is abnormally over expressed and can interact with RARA. The RNF8/RARA complex is able to promote RARA Lys48-linkage ubiquitinating degradation and block promyelocytic cell differentiation. In combination with MG132—a proteasome inhibitor—and ATRA in vitro to treat the *GTF2I*-*RARA*-positive cells, a facilitating effect of differentiation is observed. Our results indicate that RNF8 is a key target molecular for *GTF2I*-*RARA* and is responsible for ATRA resistance. Targeting of the proteasome and *RARA* receptor may provide an alternative therapeutic strategy in *GTF2I*-*RARA*-positive APL patients.

## Materials and methods

### Generation of stable cell lines expressing *GTF2I*-*RARA, RNF8, or Ub*

To generate cells stably expressing *GTF2I*-*RARA*-FLAG or control-FLAG, lentiviral vectors (*GTF2I*-*RARA*-FLAG) were transfected into HL60 cells. For details on the transfection conditions, please refer to the Additional file 1.

### Western blot and immunofluorescence staining

After cell culturing, cells were harvested and lysed in 1 × *RIPA* buffer supplemented with a protease inhibitor for western blotting. For immunofluorescence staining, fluorescent signals were acquired using a confocal microscope (Carl Zeiss AG, Oberkochen, Germany). For details on the operating steps, please refer to the Additional file 1.

### Cell viability assay

Cell viability was analyzed with methylthiazolyldiphenyl-tetrazolium bromide (MTT) assays. Briefly, cells were seeded into 96-well plates followed by the administration of ATRA treatments for 24 h, 48 h, and 72 h. After this point, 20 μl of MTT solution was transferred to each well. After incubation for 4 h, cell viability assays were performed.

### Coimmunoprecipitation

A coimmunoprecipitation (CoIP) experiment was performed as per the manufacturer’s instructions (Thermo Fisher Scientific, Waltham, MA, USA). Briefly, cell pellets were collected and lysed for 30 min on ice. Soluble lysates were incubated with an antibody coupled with resin at 4 °C overnight, and the proteins were eluted by boiling in 1 × SDS sample buffer before SDS-PAGE. The precipitated proteins were subsequently subjected to SDS-PAGE and blotted with specific antibodies.

### Cell differentiation analysis

NB4 cells and *GTF2I*-*RARA*-positive HL60 cells were treated with 1 μM of ATRA for 72 h. The cells were then incubated with PE-conjugated anti-human CD11b antibody (BioLegend, San Diego, CA, USA) and examined by flow cytometry. The percentage of CD11b-positive cells was analyzed using the FlowJo software (FlowJo LLC, Ashland, OR, USA). For morphological analysis, cytospin slides of each sample were stained with Wright-Giemsa staining and observed under an optical microscope.

### ChIP-seq

ChIP was essentially performed on FLAG-GTF2I-RARA-HL60 cells using ANTI-FLAG as per the manufacturer’s instructions (Thermo Fisher Scientific, Waltham, MA, USA). Raw data and experimental methods are available in the Additional file 1.

### Quantitative reverse transcription polymerase chain reaction

Total RNA was extracted from cells using TRIzol reagent (Takara Bio, Inc., Otsu, Japan), according to the manufacturer’s protocols. Primer sequences for *VRK2, Notch2, WDR26, ANAPC7, RNF8, EMP2, HDAC9, PTPN1, RARA*, *RXRA,* and β-actin messenger RNA (mRNA) are listed in the Additional file 1.

### In vivo ubiquitination assay

Ubiquitination of proteins requires the covalent attachment of 8.6-kDa ubiquitin (Ub) to multiple lysine residues, forming poly-Ub chains bound to target proteins, and can be seen as a ladder of high-molecular-mass species on SDS–polyacrylamide gels. For details on the experimental design, please refer to the Additional file 1.

### Luciferase assay

Transcriptional activity of *RARA* was assessed by a luciferase assay. For details on the experimental design, please refer to the Additional file 1.

### Statistical analysis

Data were expressed as mean ± standard error of the mean. Comparisons between two groups were performed by an unpaired Student’s *t* test or one-way analysis of variance test. p < 0.05 was considered statistically significant.

## Results

### *GTF2I*-*RARA* confers ATRA resistance and inhibits ATRA-induced leukemic cell differentiation

**Fig. 1:**
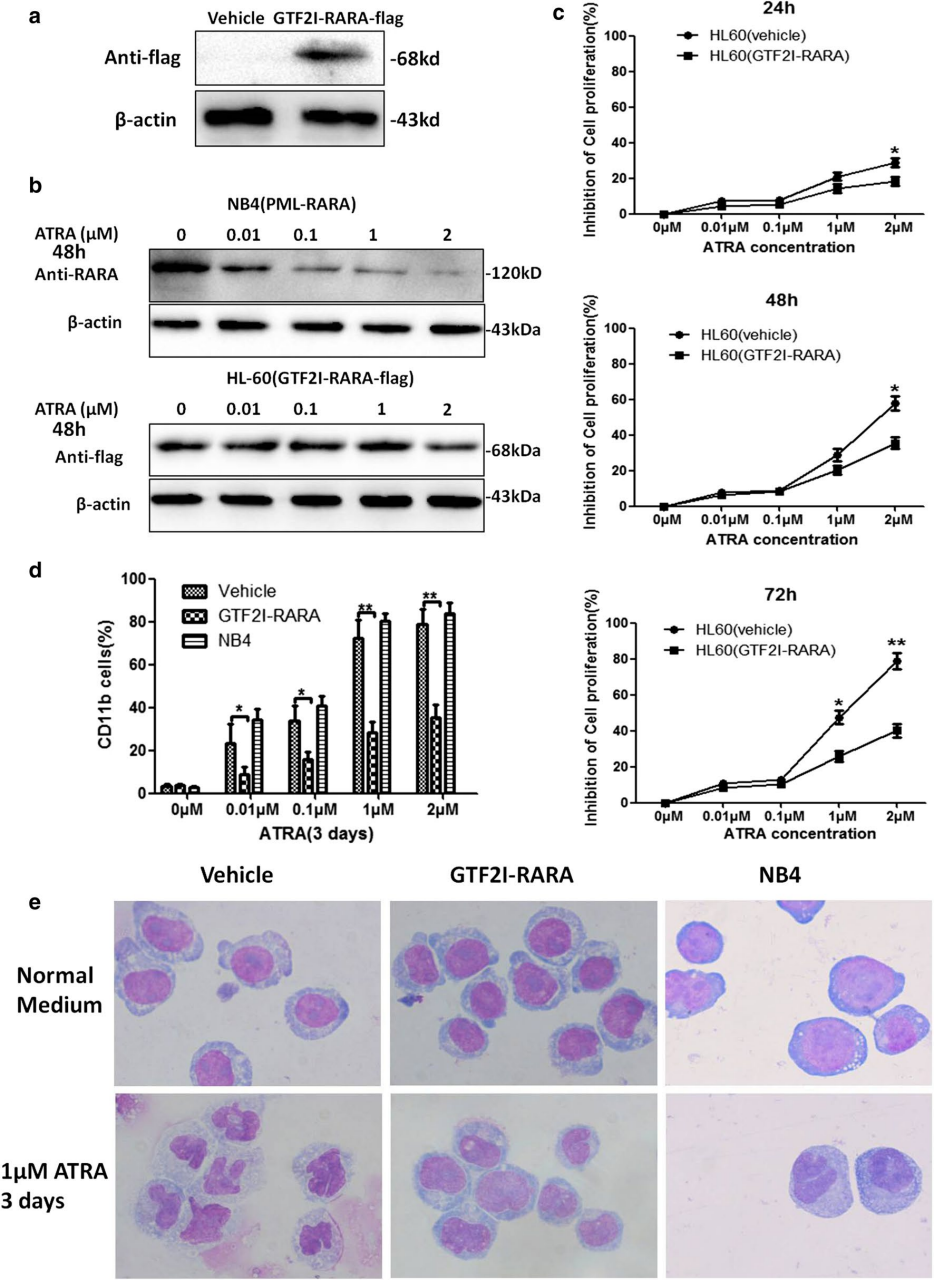
Effects of *GTF2I*-*RARA* blocking ATRA-induced differentiation and conferring proliferation potential in the HL60 cell line. **a** Western blot confirmed the stable expression of *GTF2I*-*RARA* in HL60. **b** NB4 cells or *GTF2I*-*RARA*-HL60 cells were treated with different doses of ATRA. PML-RARA or GTF2I-RARA was visualized by western blot 48 h after ATRA treatment. β-actin was used as the loading control. **c** MTT assay results for evaluating the proliferation potential in *GTF2I*-*RARA*-positive HL60 cells. Cells were exposed to different dosages of ATRA for 24 h, 48 h, and 72 h, and the proliferation-inhibiting rate is shown. The results were in the form of mean values ± standard deviations corresponding to at least three independent experiments. Statistical significance was determined with Student’s *t* test. *p < 0.05, **p < 0.01. **d** Flow cytometry analysis of cell surface CD11b in GFP-positive HL60 cells and NB4 cells at 24 h, 48 h, and 72 h after incubation in different dosages of ATRA or ethanol solvent. **e** Evaluation of morphology for cell differentiation in *GTF2I*-*RARA*-positive HL60 cells or NB4 cells 72 h after treatment with 1 μM of ATRA and staining with hematoxylin and eosin

Western blot confirmed that the *GTF2I*-*RARA* protein is stably expressed in *GTF2I*-*RARA*-positive HL60 cells (Fig. 1a). When ATRA is exposed to the *GTF2I*-*RARA*-positive HL60 and NB4 for 48 h, the *PML*-*RARA* protein expression in NB4 cells decreases in a dose-dependent manner, while there is no change regarding the effect in *GTF2I*-*RARA*-positive HL60 cells (Fig. 1b). MTT results show that ATRA might inhibit NB4 cell proliferation in a dose- and time-dependent fashion, while the *GTF2I*-*RARA*-positive HL60 cells display resistance to ATRA-mediated growth inhibition, especially when exposed to high doses of ATRA (1–2 μM) (Fig. 1c).

To check whether *GTF2I*-*RARA* impacts ATRA-induced leukemic cell differentiation, the myeloid lineage differential marker CD11b was detected by flow cytometry. Results showed that the percentage of CD11b-positive cells significantly increases in a dose-dependent manner on NB4 cells under ATRA treatment. However, *GTF2I*-*RARA*-positive cells exhibit insensitivity to ATRA-induced differentiation (Fig. 1d). Consistent with the findings of CD11b, morphological features (Wright-Giemsa staining) confirmed *GTF2I*-*RARA*-positive cells show attenuated differentiation even with ATRA treatment (Fig. 1e).

### *GTF2I*-*RARA* recruits N-CoR/SMRT/HDAC3 transcriptional corepressors and resists the dissociation of HDAC3 of ATRA induction

**Fig. 2:**
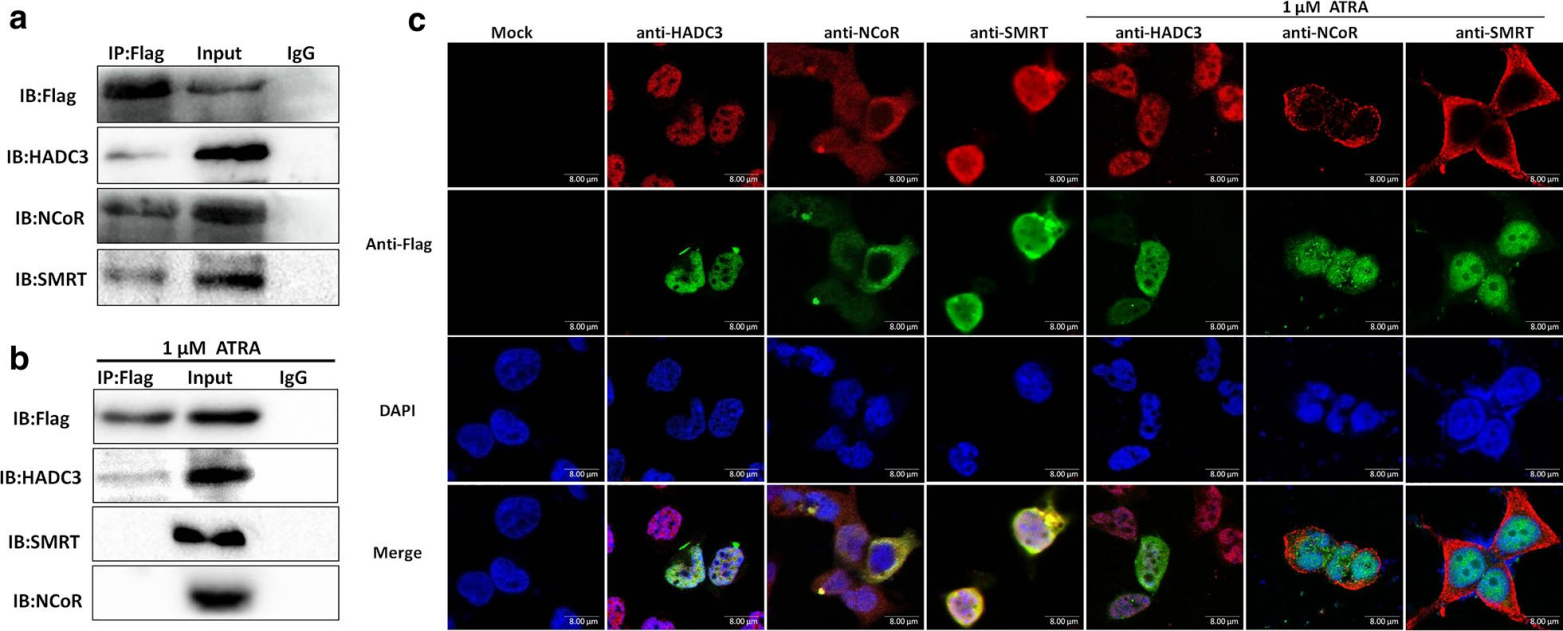
Recruitment of transcriptional corepressors by *GTF2I*-*RARA* in 293T cells. 293T cells were transfected with a FLAG-tagged *GTF2I*-*RARA* expression plasmid. **a** Coimmunoprecipitation between endogenous corepressors and *GTF2I*-*RARA*, which interacted with HDAC3, N-CoR, and SMRT. Input, non-immunoprecipitated cell lysates; IgG, control IP with isotype antibody. **b** Coimmunoprecipitation between endogenous corepressors and *GTF2I*-*RARA* in the presence of ATRA. *GTF2I*-*RARA* did not dissociate with HDAC3 but no longer interacted with SMRT and N-CoR. **c** Immunofluorescent colocalization of FLAG-tagged-*GTF2I*-*RARA* and corepressors with or without ATRA. 4′,6-diamidino-2-phenylindole (DAPI) was used for nuclear staining. At least 100 cells were counted for colocalization analysis. Scale bar = 8 μm

The *RARA* chimeric proteins commonly exhibit increased affinity for transcriptional corepressors, N-CoR/SMRT/HDAC3, which are recruited to RARE, resulting in repression of the *RARA* target gene [16]. To assess whether *GTF2I*-*RARA* could also recruit these transcriptional corepressors, we performed FLAG-tagged *GTF2I*-*RARA* pcDNA3.1 plasmid transfection and coimmunoprecipitation assays. Consistent with other *RARA* fusion proteins, *GTF2I*-*RARA* is able to be coimmunoprecipitated with N-CoR, SMRT, and HDAC3 (Fig. 2a). Immunofluorescence localization revealed that FLAG-tagged-*GTF2I*-*RARA* colocalizes with HDAC3 mainly in the nucleus and with N-CoR mainly in the cytoplasm, while it colocalizes with SMRT in both the nucleus and cytoplasm in 293T cells (Fig. 2c).

It has been shown that pharmacological doses of ATRA may precipitate the dissociation of *RARA* fusion proteins from transcriptional corepressors and re-recruit transcriptional activators [17–19]. As shown in Fig. 2b, our results indicate that a low dose of ATRA does not dissociate the *GTF2I*-*RARA*/HDAC3 complex but could impel the release of SMRT and N-CoR from the *GTF2I*-*RARA*/SMRT/N-CoR complexes. Consistent with coimmunoprecipitation, the intracellular distribution of *GTF2I*-*RARA* and corepressors changed, in that N-CoR and SMRT migrate to the cytoplasm and separate from *GTF2I*-*RARA*. Meanwhile, the GTF2I-RARA/HDAC3 complex fails to disassociate and remains colocalized in the nucleus, suggesting that HDAC3 plays a role to a certain extent in *GTF2I*-*RARA*-driven transcriptional regulation (Fig. 2c).

### Genome-wide recognition of *GTF2I*-*RARA* binding sites and differentially expressed genes

**Fig. 3:**
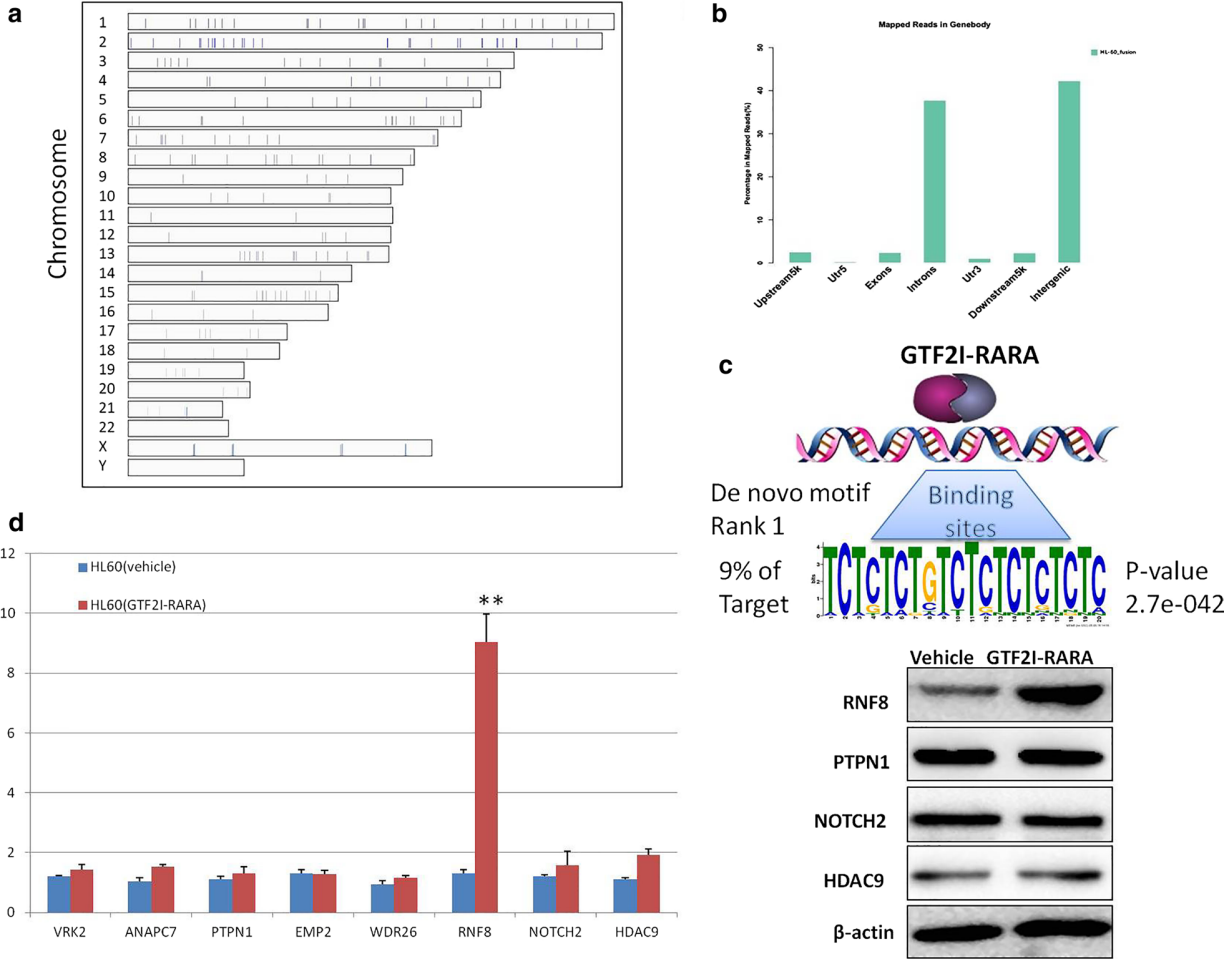
Genome-wide analysis of the *GTF2I*-*RARA* binding components in transfected HL60 cells. Binding components of *GTF2I*-*RARA* were determined using ChIP-seq. **a** Genome-wide distributions of *GTF2I*-*RARA* binding sites. **b** Distribution of binding sites of *GTF2I*-*RARA* is shown as a bar graph. **c** De novo motif logo for the top-ranking motif identified by Multiple EM for Motif Elicitation software when searching from the *GTF2I*-*RARA* binding sites. **d** Analysis of mRNA expression of potential target genes in *GTF2I*-*RARA*-transfected and control HL60 cells. **e** Western blot results for candidate targeting genes in *GTF2I*-*RARA*-transfected and control HL60 cells. **p < 0.01

To map genome-wide binding sites of GTF2I-RARA, we carried out a ChIP-seq experiment. We identified 221 binding sites of *GTF2I*-*RARA* with significant enrichment (Fig. 3a). Using Multiple EM for Motif Elicitation software, motif analysis was performed. The rank 1 de novo motif of *GTF2I*-*RARA* was detected in 9% of the DNA regions (Fig. 3c), which is not in agreement with the classic RARE. The raw data files have been uploaded to NCBI (GEO accession number: GSE122354).

While analyzing the 221 predicted peaks in the gene body, most of the peaks were found to be dispersed in introns (37.7%) and intergenic regions (42.3%), with only a few binding to exonic areas (Fig. 3b). The number of genes proximal to the *GTF2I*-*RARA* binding sites [within 5 kb upstream from the transcription start site (TSS), the gene body, and 5 kb downstream from the TTS] was 123. Known *RARA* target genes, such as *RARb2, ASB2, IL8, CEBPE,* and *PRAM1* [20–23], were not included in the 123 genes. To identify reliable novel target genes for *GTF2I*-*RARA*, we reviewed the biological function of the 123 annotated genes and focused on *VRK2, ANAPC7, PTPN1, EMP2, WDR26, RNF8, NOTCH2,* and *HDAC9* genes, which are involved in cell proliferation, differentiation, and apoptosis [24–31]. We compared the target gene expression between *GTF2I*-*RARA*-positive HL60 and control HL60 cells. Among the eight genes mentioned above, only RNF8 expression is significantly upregulated at both the mRNA and protein level (Fig. 3d, e). Because *GTF2I*-*RARA* can bind both exonic and intronic regions of RNF8 and functional RNF8 is known to act as a key Ub ligase (E3), we speculate that RNF8 might play an important role in the pathogenesis of the variant APL and confer ATRA resistance.

### *GTF2I*-*RARA* promotes interactions between RARA/RNF8 and RXRA/RNF8

**Fig. 4:**
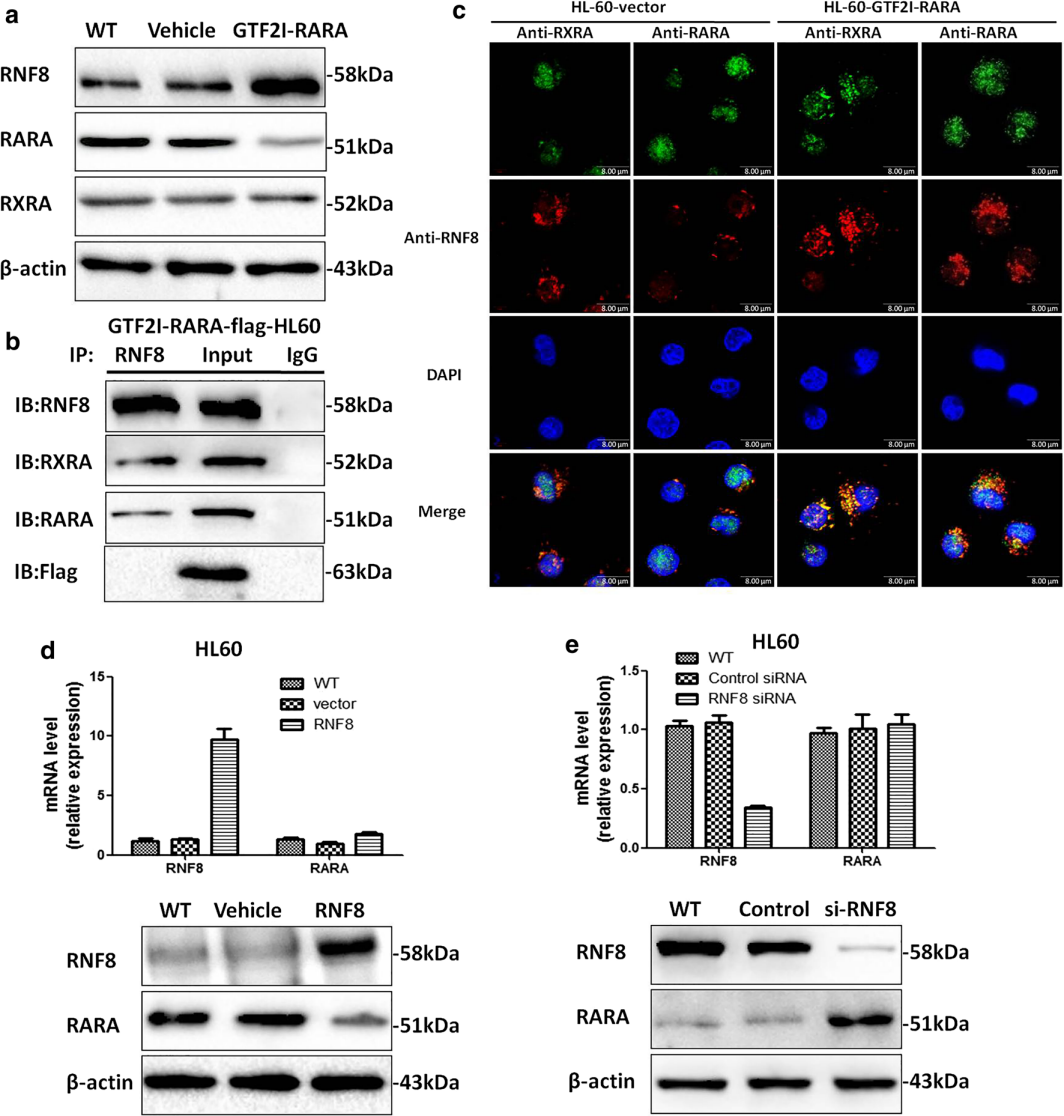
RNF8 interacts with *RARA* and downregulates *RARA* protein expression. **a** Western blot results of RNF8, *RARA,* and *RXRA* in *GTF2I*-*RARA*-positive HL60 cells. RARA expression was reduced in GTF2I-RARA-positive HL60 cells, while RXRA remain unchanged. **b** Coimmunoprecipitation assays of endogenous RARA, RXRA, GTF2I-RARA, and RNF8 in *GTF2I*-*RARA*-positive HL60 cells showed RNF8 interacted with both RARA and RXRA but had no association with GTF2I-RARA. **c** Immunofluorescent colocalization analysis of *RNF8, RARA,* and *RXRA* in *GTF2I*-*RARA*-positive HL60 cells (GFP free). DAPI was used as nuclear staining. Results indicated *GTF2I*-*RARA* might precipitate the nuclear transcript of RNF8 and the formation of RNF8/*RXRA* or RNF8/*RARA* heterodimers. At least 100 cells were counted for colocalization analysis. Scale bar = 8 μm. **d** HL60 cells were transfected with plasmid-expressed RNF8, and total mRNA and protein were extracted and assessed by qPCR and Western blot. **e** HL60 cells were transfected with siRNF8 and control siRNA, and mRNA and proteins were assessed by qPCR and western blot, respectively. The *RARA* mRNA level remained unchanged, while *RARA* protein expression significantly increased in siRNF8-HL60 cells

RNF8 is able to bind *RXRA* through the N-terminal regions of both proteins [32]. The confirmed fact that *RARA* binds to *RXRA* as a heterodimer, in turn, results in the transcriptional activation of RA target genes [33]. We presume that RNF8 likely interacts with *RARA* and participates in *RARA* ubiquitination. Our preliminary results showed that there is downregulated expression for *RARA* in *GTF2I*-*RARA*-positive HL60 cells; however, there is no change in the *RXRA* level (Fig. 4a). Coimmunoprecipitation results confirmed RNF8 not only is bound to *RXRA* but also interacts with *RARA* (Fig. 4b), suggesting that RNF8 can form a heterodimer with *RARA* or *RXRA* and regulate transcriptional activation or inhibition of RA targeting genes. The intracellular localization results for RNF8, *RXRA,* and *RARA* proteins revealed that RNF8 is predominantly distributed in the cytoplasm, while *RXRA* and *RARA* are localized both in the nucleus and cytoplasm (Fig. 4c, left panel). Furthermore, in *GTF2I*-*RARA*-positive HL60 cells, RNF8 displays overt co-localization with *RXRA* or *RARA* mainly in the cytoplasm (Fig. 4c, right panel), suggesting that *GTF2I*-*RARA* mediates the transposition of RNF8 and the formation of RNF8/RXRA or RNF8/RARA heterodimers. To prove whether RNF8 disturbs *RARA* expression by downregulation, RNF8-overexpressed or knocked down HL60 cell models were constructed. Results showed that the protein level, not the mRNA level of RARA, is downregulated in RNF8-overexpressed HL60 cells. Meanwhile, RNF8-siRNA HL60 cells exhibit upregulated *RARA* protein expression and have no effect on RARA mRNA expression (Fig. 4d, e). These results confirm that RNF8 is a negative control factor for the *RARA* protein and might influence the post-translational modification of RARA. This could be responsible for *GTF2I*-*RARA*-driven ATRA resistance.

### RNF8 triggers Lys48-linkage Ub modification on RARA and contributes to ATRA-induced differentiation arrest

**Fig. 5:**
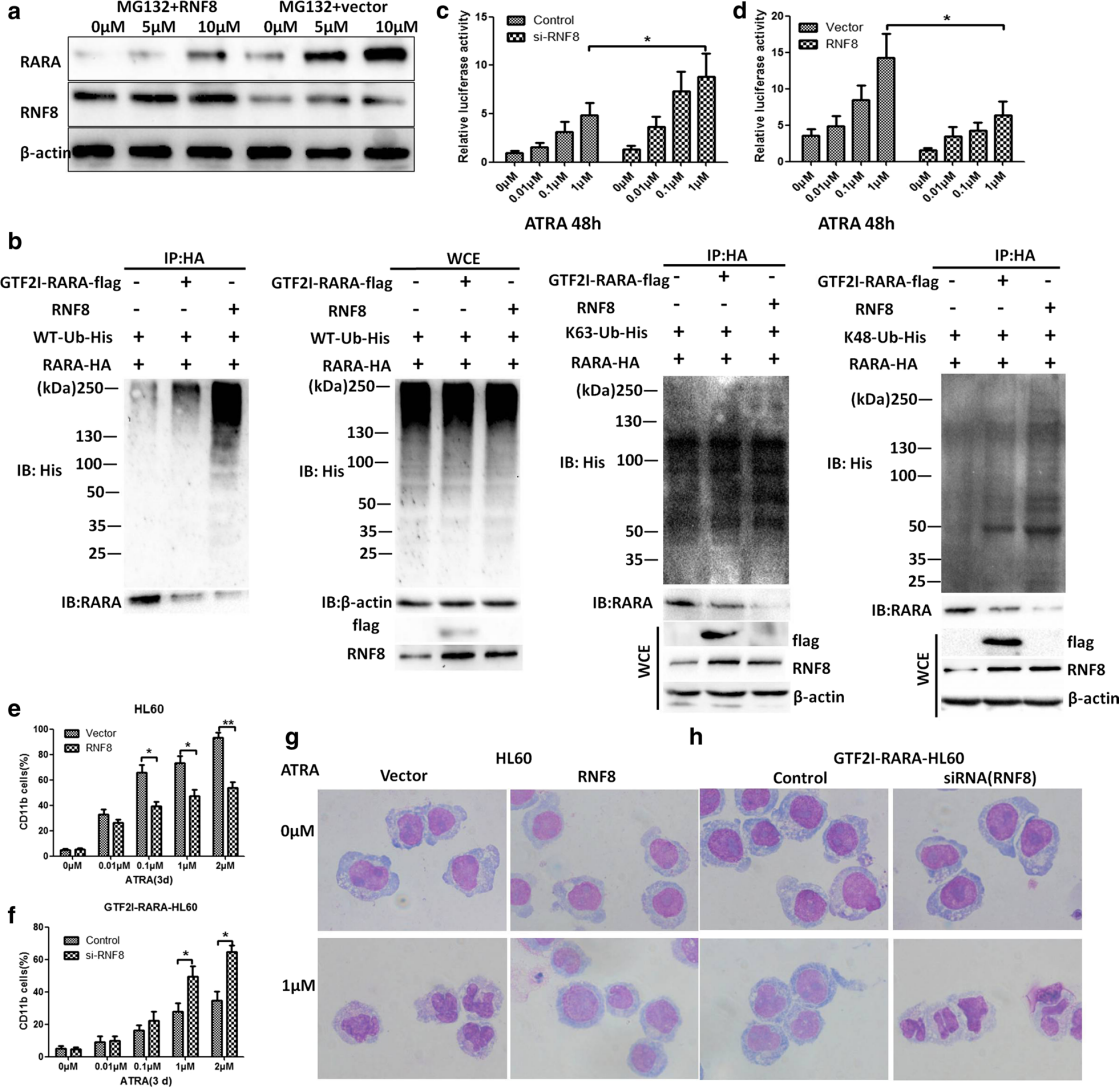
RNF8 triggers RARA Lys48-linkage polyubiquitination and inhibits ATRA-induced differentiation. **a** During western blot analysis, HL60 cells were separately transfected with RNF8 expressing plasmid or empty vector and treated with 0 μM, 5 μM, and 10 μM MG132 for 4 h, with results indicating MG132 could reverse RNF8-mediated *RARA*-expressing inhibition. **b** Linkage-specific ubiquitination on RARA in response to overexpression of RNF8 or GTF2I-RARA. Coimmunoprecipitation following co-overexpression of *RNF8*, *RARA*-Ha, *Ub*-His/K63-*Ub*-His/K48-*Ub*-His, and *GTF2I*-*RARA* in 293T. RARA and its post-translational derivatives were purified by an HA antibody, and linkage-specific ubiquitinated RARA was detected by immunoblot using a His antibody. **c** 293T cells were cotransfected with the RARE reporter genes and plasmid encoding RNF8 or empty vector. Relative luciferase activity of each group in response to various concentrations of ATRA is shown in the histogram. **d** 293T cells were cotransfected with the RARE reporter genes and siRNF8. Relative luciferase activity in response to various concentrations of ATRA is shown in the histogram. **e**, **f** HL60 cells were transfected with a plasmid encoding RNF8. The CD11b differentiation marker was assessed by flow cytometry under exposure to various concentrations of ATRA for 3 days. For morphological analysis, each sample was stained with Wright-Giemsa and observed under an optical microscope (×1000) after 1 μM of ATRA treatment for 3 days. **g**, **h** *GTF2I*-*RARA*-HL60 cells were transfected with siRNF8. CD11b differentiation marker assay and Wright-Giemsa staining procedures were completed in the same manner as described above. Error bars represent the mean of experiments in triplicate. *p < 0.05, **p < 0.01. WCE: whole cell extracts

RNF8 plays a critical role in catalyzing both K48- and K63-linked Ub chains. While functions of many of these distinct Ub chains remain obscure, polyubiquitin chains composed of K48-linkages are generally associated with commitment for proteasomal degradation, whereas K63-linked polyubiquitylation plays established roles in DNA damage–repair, protein kinase activation, and receptor endocytosis [29, 34]. We presume that RNF8 might also be involved in *RARA* ubiquitination. To validate this assumption and further illustrate the ubiquitin landscape on RARA, we first used the proteasome inhibitor MG132 at different concentrations and applied it to HL60 cells expressing RNF8. Results showed that RNF8 overexpression could antagonize MG132-induced *RARA* expression (Fig. 5a). To investigate linkage-specific polyubiquitin conjugation on RARA, we further performed coimmunoprecipitation in 293T cells following co-overexpression of *RNF8*, *RARA*-Ha, *GTF2I*-*RARA,* wild type (WT)-*Ub*-His, or lysine-only mutant (K48, K63) Ub, in which all lysine residues except one were mutated to arginine. We found that overexpression of *RNF8* or *GTF2I*-*RARA* leads to a marked increase in *RARA* Lys48-linkage Ub modification, as well as Ub modification, but it has a minimal effect on K63-linked ubiquitination of RARA (Fig. 5b). These results support the idea that RNF8 is responsible for K48-ubiquitin chain formation of RARA and has been associated with RARA proteasomal degradation. It is known that *RARA* plays a critical role in myeloid differentiation. Hence, we further assessed the effects of RNF8 on the transcriptional activity of *RARA*. Luciferase assay results indicated that RNF8 could significantly suppress the transcriptional activity of RARE (Fig. 5c), while knockdown of RNF8 by siRNF8 exhibits an ATRA-induced transcriptional activation in *GTF2I*-*RARA*-positive 293T cells (Fig. 5d). To uncover whether overexpression of RNF8 could counteract ATRA-induced cell differentiation, *RNF8*-transduced HL60 cells were treated with various concentrations of ATRA, and CD11b was detected by flow cytometry. The results showed that the proportion of CD11b-positive cells is found at a lower level in *RNF8*-transduced HL60 cells compared with the control group (Fig. 5e). Wright-Giemsa staining also confirmed that HL60 with RNF8 overexpression represents attenuated ATRA-induced differentiation (Fig. 5g). However, knockdown of RNF8 in *GTF2I*-*RARA*-positive HL60 cells increases the percentage of CD11b-positive cells and confers sensitivity of differentiation to ATRA (Fig. 5f). Cell morphological observation showed that downregulated RNF8 reverses the effects of differentiation arrest of *GTF2I*-*RARA* on ATRA (Fig. 5h).

### MG132 possesses synergistic effects in ATRA-induced *GTF2I*-*RARA*-positive cell differentiation

**Fig. 6:**
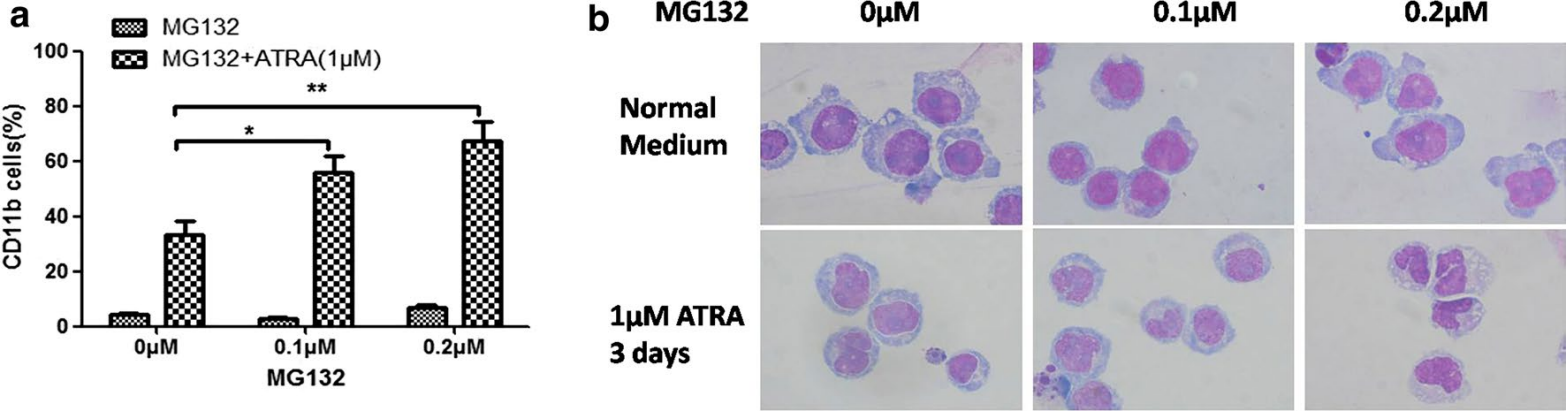
MG132 combined with ATRA induced *GTF2I*-*RARA*-positive cell differentiation. *GTF2I*-*RARA*-positive HL60 cells were treated with various concentrations of MG132 and 1 μM of ATRA for 3 days. **a** The myeloid differentiation marker CD11b was assessed by flow cytometry. The percentage of CD11b-positive cells increased in a dose-dependent manner on the cells treated with MG132 in combination with ATRA. Results are represented in the form of mean values ± standard deviations from three independent experiments. *p < 0.05, **p < 0.01. **b** When *GTF2I*-*RARA*-positive cells were exposed to ATRA (1.0 μM) and MG132 (0.2 μM), they manifested distinctly different characteristics (×1000)

Based on the results above, we envisage that inhibiting *RARA* degradation might help to restore sensitivity to ATRA in *GTF2I*-*RARA*-positive HL60 cells. Therefore, we treated *GTF2I*-*RARA*-positive HL60 cells with both MG132 (0–0.2 μM) and ATRA (1.0 μM) and observed cell differentiation status. Our results showed that the proportion of CD11b-positive cells is significantly increased in the MG132 + ATRA group compared with ATRA alone (Fig. 6a). Additionally, cell morphology revealed that *GTF2I*-*RARA*-positive cells possess differentiated characteristics following the progressive increases of MG132 concentration (Fig. 6b).

## Discussion

We call attention to our previous findings regarding a variant APL patient with t(7; 17) in whom a novel *GTF2I*-*RARA* fusion was determined [15]. Just as *ZBTB16*-*RARA* or *STAT5b*-*RARA* fusions exist, this new subtype of APL manifested insensitivity to ATRA and conventional chemotherapy and had a poor prognosis. It has been shown that the nature and gene structures of the fusion partners have decisive impacts on the disease state and therapeutic response of ATRA [2]. In this study, we constructed a cell line expressing *GTF2I*-*RARA,* certified the phenotype of ATRA resistance, and further probed into the molecular mechanisms for ATRA resistance and transcriptional regulation arising from *GTF2I*-*RARA*.

It has been confirmed that ATRA can induce APL cell differentiation by promoting the fusion protein to undergo degradation [35, 36]. Meanwhile, ATRA can interact with *RARA*, leading to ligand-dependent transactivation of target genes, and induce APL cell differentiation; additionally, overdose of ATRA further promotes APL cell apoptosis [37, 38]. In order to investigate the mechanism and resistant phenotype of *GTF2I*-*RARA*, we established a *GTF2I*-*RARA* stably expressed cell line by lentivirus infection. We showed that when a therapeutic dose of ATRA was used, *GTF2I*-*RARA* protein levels did not change in *GTF2I*-*RARA*-positive cells, suggesting that ATRA could not degrade *GTF2I*-*RARA*. As *GTF2I*-*RARA*-positive cells were exposed to different doses of ATRA, an effect of ATRA-mediated growth inhibition was attenuated, and ATRA-induced cell differentiation action was blocked. These results indicate that *GTF2I*-*RARA* is different from the typical PML/*RARA*, which displays ATRA resistance and differentiation arrest. Moreover, the GTF2I-RARA-positive cell model greatly mimics the clinical therapeutic response seen with our earlier patient and paves the way for further work. Results from Lin et al. showed TBLR1-*RARA* fusion can recruit transcriptional corepressors, including N-CoR, SMRT, and HDACs [39]. N-CoR/SMRT can recruit HDAC3 to enhance its activity for transcriptional repression [40]. Most *RARA* fusion proteins repress transcription to a greater extent than *RARA* and block myeloid differentiation at the promyelocyte stage [38, 39]. Only pharmacological doses of ATRA may dissociate transcriptional corepressors and recruit transcriptional coactivators, resulting in the differentiation of APL cells [16]. *GTF2I* is reported to recruit corepressor complexes such as HDAC1, HDAC3, LSD1, and PRC [41–44], and the recruitment is associated with the conserved N-terminal leucine zipper (LZ) (amino acids 23–44) of *GTF2I* [13]. In our study, we found that *GTF2I*-*RARA* was also associated with N-CoR, SMRT, and HDAC3. However, when exposed to 1 μM of ATRA for 48 h, *GTF2I*-*RARA* released N-CoR and SMRT but did not influence the interaction between HDAC3 and *GTF2I*-*RARA*, suggesting that the *GTF2I*-*RARA*-HDAC3 complex contributes to the repression of *RARA* target genes and confers ATRA resistance. The N-terminal leucine zipper (amino acids 23–44) on *GTF2I* mediates homo- or heteromeric interaction [13]. In the *GTF2I*-*RARA* fusion protein, the first 195 amino acids of *GTF2I*, including the LZ and the first I-repeat (R1, 104–176), are retained, providing the possibility of *GTF2I*-*RARA*/HDAC3 multimerization.

Using state-of-the-art ChIP-seq and ChIP-on-chip technologies, some groups have shed light on the majority of direct downstream targets of PML-RARA [9, 10]. To determine the essential downstream target genes of *GTF2I*-*RARA*, we performed ChIP-seq to search for key target genes that interact with *GTF2I*-*RARA*. Among the identified 123 peaks relative to annotative genes, we discovered *RNF8* was significantly upregulated at both the mRNA and protein level and speculated that *RNF8* may be an important target gene of *GTF2I*-*RARA*. *RNF8* belongs to the member of the RING-type subfamily and encodes an E3 Ub ligase. *RNF8* plays a critical role in transducing DNA damage signals, interacting with E2s, and catalyzing both K48- and K63-linked Ub chains [45]. Polyubiquitin chains, mainly consisting of K48 linkages, are associated with proteasomal degradation. Different E3 Ub ligases can bind to relevant substrate proteins. RNF8 could act as a regulator of *RXRA*, by mediating transcriptional activity through interaction between respective N-terminal regions [32]. Our results demonstrated that RNF8 interacts with both *RARA* and *RXRA* and that RNF8 colocalizes with *RXRA* and *RARA*. In *GTF2I*-*RARA*-positive cells, an upregulated RNF8 protein expression coupled with a downregulated *RARA* protein level suggests a mechanism in which RNF8 may mediate the degradation and transcriptional activation of *RARA*.

*RARA* and *RXRA* can form heterodimers, which transduce signals by binding to RARE in the promoter regions of target genes [2]. We revealed that overexpression of RNF8 in HL60 cells attenuates the transcriptional activity of the RARE reporter gene. When RNF8 was knocked down in *GTF2I*-*RARA*-positive cells, transcriptional activity increases, and the sensibility of ATRA-induced cell differentiation is regained. Consistent with luciferase assays, overexpressed RNF8 decreases the sensitivity of HL60 cells to ATRA and blocks cell differentiation, indicating that RNF8 plays an important role in impeding cell differentiation and ATRA resistance.

It has been reported that RNF8 catalyzes Ub chain formation via differential RING-dependent interactions with its E2 s. RNF8 overexpression may facilitate *RARA* ubiquitination. Our data revealed that *RARA* Lys-48 specific ubiquitination is enhanced in *GTF2I*-*RARA/*RNF8-overexpressed cells, which indicates that the RNF8-dependent K48-ubiquitin chains may lead to downregulated RARA protein levels. Using MG132 in combination with ATRA, we observed a synergistic effect on ATRA-induced *GTF2I*-*RARA*-positive cell differentiation, suggesting that the inhibition of *RARA* ubiquitination contributes to reverse the ATRA resistance of *GTF2I*-*RARA*-positive cells.

**Fig. 7:**
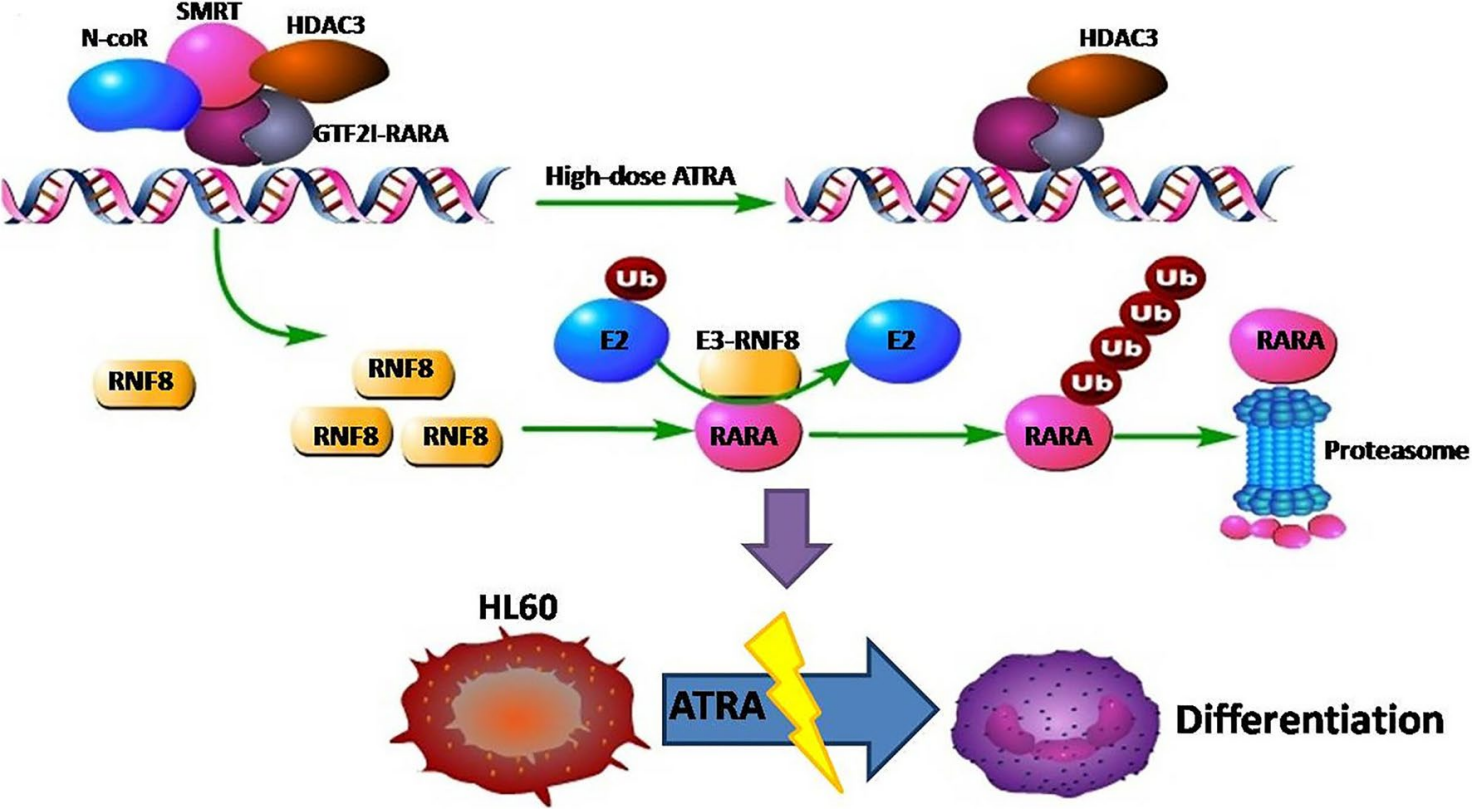
Schematic diagram of the *GTF2I*-*RARA*-driven ATRA resistance mechanism

Based on our results, a model of an ATRA-resistance mechanism on *GTF2I*-*RARA*-positive cells is outlined in Fig. 7. As a transcriptional factor, *GTF2I*-*RARA* fusion selectively binds to RNF8 and upregulates the expression of RNF8. RNF8 interacts with *RARA* and promotes *RARA* ubiquitination and degradation. As a result, the transcriptional activity of *RARA* is impaired, leading to the blocking of cell differentiation and ATRA resistance.

## Conclusion

This study reveals the mechanism of *GTF2I*-*RARA*-driven ATRA resistance and identifies potential target genes involved in cell differentiation regulation. ATRA resistance on *GTF2I*-*RARA*-positive cells is mainly attributed to upregulated RNF8 expression, which could lead to RARA degradation. Aberrant recruitment of HDAC3 by *GTF2I*-*RARA* may play a minor role. Moreover, combining MG132 and ATRA can increase the sensitivity of ATRA-induced cell differentiation on *GTF2I*-*RARA*-positive cells and reverse ATRA tolerance. In conclusion, these findings provide us new therapy targets to reverse the ATRA resistance of GTF2I-RARA.

## Additional file

**Additional file 1.** Name of genes proximal to the GTF2I-RARA binding sites and supplementary instruction for the experimental methods.

## Abbreviations

APL: acute promyelocytic leukemia
RNF8: RING finger protein 8
ATRA: *all*-*trans* retinoic acid
ATO: arsenic trioxide
ChIP-seq: ChIP-sequencing
CoIP: coimmunoprecipitation
mRNA: messenger RNA
