# Supplementary material for: RNF8 is responsible for ATRA resistance in variant acute promyelocytic leukemia with GTF2I/RARA fusion, and inhibition of the ubiquitin–proteasome pathway contributes to the reversion of ATRA resistance
Source: Cancer Cell Int. 2019 Apr 4;19:84. doi: 10.1186/s12935-019-0803-4 (PMC6449960; doi:10.1186/s12935-019-0803-4)
Supplement: Supplementary file 1 — Additional file 1. Name of genes proximal to the GTF2I-RARA binding sites and supplementary instruction for the experimental methods. [file 12935_2019_803_MOESM1_ESM.docx]

Additional file 1

This file has been provided by the authors to give readers additional information about their work.

Supplement to: Wenzhe Yan, Ji Li, Yang Zhang L, et al. RNF8 is responsible for ATRA resistance in variant acute promyelocytic leukemia with GTF2I/RARA fusion, and inhibition of the ubiquitin-proteasome pathway contributes to the reversion of ATRA resistance

Table S1 Genes proximal to the GTF2I-RARA binding sites

| Element | Genes_name |
| --- | --- |
| Upstream5k | LINC01226 |
| Upstream5k | SPG21 |
| Upstream5k | LINC00273 |
| Upstream5k | KRTAP9-6 |
| Upstream5k | IGFBP5 |
| Upstream5k | MIR3648-1 |
| Utr5 | RNF8 |
| Exons | WDR26 |
| Exons | PARG |
| Exons | PRC1 |
| Exons | C17orf105 |
| Exons | KDSR |
| Exons | STON1-GTF2A1L |
| Exons | SAMSN1 |
| Exons | RNF8 |
| Introns | CA6 |
| Introns | TINAGL1 |
| Introns | ERI3 |
| Introns | TRABD2B |
| Introns | HFM1 |
| Introns | TGFBR3 |
| Introns | NOTCH2 |
| Introns | ARHGEF11 |
| Introns | CACNA1E |
| Introns | PTPRC |
| Introns | NENF |
| Introns | TAF5L |
| Introns | LYST |
| Introns | BTRC |
| Introns | DLG2 |
| Introns | PDE3A |
| Introns | LOC643770 |
| Introns | UHRF1BP1L |
| Introns | ANAPC7 |
| Introns | RNASEH2B |
| Introns | PIBF1 |
| Introns | KLF12 |
| Introns | MIR4500HG |
| Introns | PPP2R3C |
| Introns | TC2N |
| Introns | IGDCC3 |
| Introns | THSD4 |
| Introns | PRC1-AS1 |
| Introns | PRC1 |
| Introns | LINC00923 |
| Introns | EMP2 |
| Introns | GNAO1 |
| Introns | LOC440434 |
| Introns | ERN1 |
| Introns | ARSG |
| Introns | C18orf25 |
| Introns | NCAN |
| Introns | SNTG2 |
| Introns | SNTG2 |
| Introns | VIT |
| Introns | MTA3 |
| Introns | ABCG8 |
| Introns | PPM1B |
| Introns | ASB3 |
| Introns | VRK2 |
| Introns | VPS54 |
| Introns | ANKRD30BL |
| Introns | ANKRD30BL |
| Introns | ARHGAP15 |
| Introns | ARHGAP15 |
| Introns | STK39 |
| Introns | LRP2 |
| Introns | GULP1 |
| Introns | MYO1B |
| Introns | PLCL1 |
| Introns | PID1 |
| Introns | PTPN1 |
| Introns | NTSR1 |
| Introns | MIR5009 |
| Introns | C3orf20 |
| Introns | ZNF385D |
| Introns | CGGBP1 |
| Introns | CPOX |
| Introns | GRAMD1C |
| Introns | CNBP |
| Introns | TMCC1 |
| Introns | MBNL1 |
| Introns | RBM47 |
| Introns | CAMK2D |
| Introns | LARP1B |
| Introns | IRF2 |
| Introns | SKIV2L2 |
| Introns | MCTP1 |
| Introns | EFNA5 |
| Introns | KCNN2 |
| Introns | NSD1 |
| Introns | GMDS |
| Introns | FARS2 |
| Introns | RNF8 |
| Introns | ZFAND3 |
| Introns | ZFAND3 |
| Introns | MED23 |
| Introns | HBS1L |
| Introns | PEX7 |
| Introns | LOC100507557 |
| Introns | MAP3K44 |
| Introns | SDK1 |
| Introns | ANKMY2 |
| Introns | AHR |
| Introns | HDAC9 |
| Introns | NUDCD3 |
| Introns | CALN1 |
| Introns | RSBN1L |
| Introns | LMBR1 |
| Introns | CSMD1 |
| Introns | SLCO5A1 |
| Introns | CPQ |
| Introns | STK3 |
| Introns | CSMD3 |
| Introns | FBXO32 |
| Introns | PYCRL |
| Introns | LINGO2 |
| Introns | SEMA4D |
| Introns | PALM2-AKAP2 |
| Utr3 | WDR26 |
| Utr3 | KDSR |
| Downstream5k | DIO1 |
| Downstream5k | MIR3648-1:--;MIR3687-1:-- |
|  |  |

**Materials and Methods**

**Cell lines and primary cultures**

**Human leukemia NB4 cells, HL-60 cells, and 293T cells were retained in our laboratory. NB4 cells and HL-60 cells were cultured and maintained in 1640 medium (HyClone, Logan, UT, USA) supplemented with 10% fetal bovine serum (BI, Israel) under 5% carbon dioxide. 293T cells were cultured and maintained in Dulbecco's modified Eagle's medium (HyClone Laboratories, Logan, UT, USA) supplemented with 10% fetal bovine serum under 5% carbon dioxide.**

**Plasmid constructs**

**Full-length cDNA of *GTF2I-RARA* was obtained by reverse transcription polymerase chain reaction (PCR) from the patient’s primary cells and identified by Sanger sequencing. The cDNA of *GTF2I-RARA* was cloned into both pcDNA3.1 expression vector with FLAG-tag fused to the carboxy termini of *GTF2I-RARA* and pGC-FU-3FLAG-SV40-EGFP-IRES-puromycin** **lentiviral vector. Full-length cDNA of wild-type *RARA* was cloned into pDsRED2-N1-hemagglutinin (HA) plasmid; full-length cDNA of wild type *RNF8* was cloned into pcDNA3.1(-); and full-length cDNA of ubiquitin (Ub) was cloned into pcDNA3.1/myc-his(-)a. Corresponding empty vectors were also constructed. All plasmid constructs were performed at Genechem Co., Ltd. in Shanghai, China.**

**The primer sequences used were as follows**

**Sense: 5’-ATGCTGCGGTGGTTGTGTG-3’, Antisense: 5’-CAGAAGGAGCCCATTTAAGCAC-3’ for *VRK2*; Sense: 5’-AAGCAGCCAAGATCCTGTTAGAC-3’, Antisense: 5’-CCACAGATGACAGGTGAGAGAGC-3’ for *Notch2*; Sense: 5’-AAGTTCTACGCTGTGAATTGACG-3’, Antisense: 5’-CACTGATGGTGGTAAATAGGTCTG-3’ for *WDR26*; Sense: 5’-CAAAACATTATTAGATAAAGCCCTGAC-3’, Antisense: 5’-TCTGATTAGCCAGTGCGTTCC-3’ for *ANAPC7*; Sense: 5’-GCCCGGCTTCTTCGTCAC-3’, Antisense: 5’-GACACCAAATCCTCGTCCTACAG-3’ for *RNF8*; Sense: 5’-CTGCTAGTGGTGGGTGGAAGA-3’, Antisense: 5’-CTGGCACGCAGGTTAAGGAT-3’ for *EMP2*; Sense: 5’-GCGGAAGGATGGAAATGTTG-3’, Antisense: 5’-TGAATTAGAATGCGTTGCTGTG-3’ for *HDAC9*; Sense: 5’-AAGCAGCCAAGATCCTGTTAGAC-3’, Antisense: 5’-CCACAGATGACAGGTGAGAGAGC-3’for *PTPN1*; Sense: 5’-ACCCCCTCTACCCCGCATCTACAAG -3’, Antisense: 5’-CATGCCCACTTCAAAGCACTTCTGC-3’ for *RARA*; Sense: 5’-AGATGGACAAGACGGAGCTG-3’, Antisense: 5’-CCAAGGACGCATAGACTTC-3’ for *RXRA*;**

**Sense: 5’-TTCCAGCCTTCCTTCCTGGG-3’, Antisense: 5’-TTGCGCTCAGGAGGAGCAAT-3’ forβ-actin.**

**Transfection**

**Lentiviral vector containning GTF2I-RARA-flag was added into HL60 cell medium. After 72 hours of transfection, stable transfectants were selected in medium containing 3 mg/ml of puromycin for 3 days. After 2 to 3 passages in the presence of puromycin, the cultured cells were sorted using the BD FACSCalibur™ system (BD Biosciences, Franklin Lakes, NJ, USA) to identify GFP-positive cells to be used for the next experiments without the need for cloning. To generate cells expressing RNF8 or si-RNA, plasmids or si-RNF8 was transfected into HL-60 cells by Nucleofector™ Kits for HL-60 (Lonza Group, Basel, Switzerland) according to the manufacturer’s instructions. To generate cells expressing GTF2I-RARA-FLAG, RNF8, Ub and lysine-only mutant(K48, K63) Ub, in which all lysine residues except one were mutated to arginine, plasmid were transfected into 293T through transfection reagent(Biotool, USA). After 24 hours of transfection, transfectants were then used for further experiments without cloning.**

**Western blot and immunofluorescence staining**

**Total protein (50 µg) were fractionated by sodium dodecyl sulphate polyacrylamide gel electrophoresis (SDS-PAGE) and transferred to PVDF membrane. Immunoblot analysis was performed with antibodies listed in the Online Supplementary Methods. Signals were detected by ChemiDoc™ XRS+ imaging system (Bio-Rad Laboratories, Hercules, CA, USA). β-actin was used as a loading control. For immunofluorescence staining, cells were seeded on glass coverslips and transfected as indicated. Twenty-four hours post-transfection, cells were fixed in 4% buffered paraformaldehyde for 15 minutes at room temperature, permeabilized with 0.5% Triton X-100 for 15 minutes, and blocked with 5% BSA for 30 minutes at 37°C. The primary antibodies HDAC3, N-CoR, and SMRT (Santa Cruz Biotechnology, Inc., Dallas, TX, USA) were used. Fluorescent signals were acquired using a confocal microscope (Carl Zeiss AG, Oberkochen, Germany).**

**ChIP-seq**

**Briefly, approximately 6×10^6^ *GTF2I-RARA*-FLAG-HL-60 cells were used for the ChIP procedure. Cells were cross-linked with 1% formaldehyde for 10 minutes at 25°C, which was followed up with by the addition of 10× glycine to terminate cross-linking. Cells were then treated with cell lysis buffer including cocktail protease inhibitor to solubilize the chromatin. Lysates were digested by micrococcal nuclease to ensure the size of DNA fragments ranged from 100 bp to 500 bp. Chromatin was incubated with anti-FLAG antibody (ChIP grade; Cell Signaling Technology, Inc., Danvers, MA, USA) coupled to protein A/G agarose for 12 hours and then harvested. Ten percent digested original chromatin was used as an input.**

**A ChIP-seq library was prepared from approximately 20 ng of ChIP-DNA and an input sample from a mixture of 3 ChIP operations. The concentration and integrity of the DNA sample was checked by gel electrophoresis. The selected libraries for a fragment size of 100 bp to 500 bp were amplified using PCR. ChIP-seq libraries were run on a HiSeq next-generation sequencer (Illumina, San Diego, CA, USA). After abandonment of polluted reads, low-quality reads, and reads with N > 5%, sequenced-50 base short single reads were mapped to the human genome, and the peaks were identified by Model-based Analysis of ChIP-Seq S14 software.**

**Small interfering RNA**

**HL-60 cells were plated into 6-well plates and transfection procedures were carried out according to the manufacturer’s protocols. Three different *RNF8* small interfering RNA (siRNA) sequences were tested for screening the efficacy of RNF8 knock-down. *RNF8* siRNA-1 (5’-GGACAATTATGGACAACAA-3’) was chosen for the RNA interference experiment. Before each experiment, we transiently transfected HL-60 cells using Nucleofector™ Kits for HL-60 (Lonza Group, Basel, Switzerland). siRNA or control RNA for each transfection were designed and synthesized by RiboBio in Guangzhou, China. The results are the means of 3 independent experiments.**

**In vivo ubiquitination assay**

**293T cells were transfected with various combinations of plasmids encoding RNF8, *RARA*-HA, and Ub-His/K48-Ub-His/K63-Ub-His expression constructs, as described above. At 12 hours post-transfection, cells were incubated with 10 mM MG132 (MedChemExpress, Monmouth Junction, NJ, USA), a potent proteasomal inhibitor, for** **4 hours prior to protein extraction. *RARA* was immunoprecipitated using anti-HA antibody (Cell Signaling Technology, Inc., Danvers, MA, USA) and subjected to SDS-PAGE, and ubiquitinated *RARA* was analyzed by Western blotting using anti-His antibody (Cell Signaling Technology, Inc., Danvers, MA, USA). The overall ubiquitination of protein in the whole cell extract was used as an internal control.**

**Luciferase assay**

**A combination of retinoic acid response element (RARE) Cignal reporter (Cignal™ Pathway Reporter Kit, Qiagen, Hilden, Germany), including inducible transcription factor responsive construct and constitutively expressing Renilla luciferase construct, and respective vectors (*GTF2I-RARA*, *RNF8* expression vectors) were transiently cotransfected into 293T cells. The cells were then incubated with different concentrations of ATRA for 48 hours. The luciferase assay was performed using a Dual-Luciferase® Reporter Assay System (Transgene, Illkirch-Graffenstaden, France) according to the manufacturer’s instructions and detected by** **multimode plate reader (PerkinElmer, Inc., Waltham, MA, USA). The ratio between firefly and Renilla luciferase was used to normalize the transfection efficiency. The results are the means of 3 independent experiments.**
